# Heat Transfer in Nanostructured Materials

**DOI:** 10.3390/nano13061062

**Published:** 2023-03-15

**Authors:** Ming-Hui Lu

**Affiliations:** 1 National Laboratory of Solid State Microstructures & College of Engineering and Applied Sciences, Nanjing University, Nanjing 210093, China; luminghui@nju.edu.cn; 2 Jiangsu Key Laboratory of Artificial Functional Materials, Nanjing University, Nanjing 210093, China

Thermal manipulation has garnered considerable attention for its potential applications in diverse areas, including microelectronics, thermal logic devices, and thermoelectrics. Manipulating heat transfer in nanostructured materials is particularly desirable, especially for structures with high thermal conductivity used in thermal dissipation. However, the demand for high-power micro/nano chips exceeds the capabilities of even cutting-edge thermal management technologies, which currently represent a bottleneck in further development. Conversely, structures with low thermal conductivity are of interest in various areas such as thermal barriers and thermoelectrics. Understanding heat transfer is thus a fundamental problem that requires comprehensive study. However, studying heat transfer in nanostructures presents significant challenges due to the dominance of interfaces, boundaries, and imperfections in its behavior, and the underlying physical mechanisms remain unclear [1].

This Special Issue, entitled “Heat Transfer in Nanostructured Materials”, provides a platform for presenting original and review articles that showcase recent advances in heat transfer within low-dimensional materials and nanofluids. The issue will systematically introduce and discuss the design, construction, characterization, and potential applications of these structures.

Low-dimensional structures have demonstrated significant potential for nanoscale dissipation as thermal interface materials (TIMs). Lv et al. [2] developed a novel approach to enhance the thermal conductivity of graphene papers by depositing AgNWs on graphene sheets, resulting in a highly promising TIM with a cross-plane thermal conductivity of up to 7.48 W m^−1^ K^−1^. Li et al. [3] revealed size-dependent thermal conductivity in VO_2_ nanowires that showed an increase in jump during metal-insulator transition temperature with increasing sample thickness. Phonons were identified as major carriers that dominate thermal transport in the nanowires. Jiang et al. [4] investigated the issue of lattice thermal conductivity calculations for two-dimensional (2D) materials and found that hydrodynamic phonon transport plays a crucial role in predicting thermal conductivity in 2D materials such as graphene and silicene. However, this phenomenon was not observed in bulk silicon. Wieser et al. [5] examined the impact of organic linkers, inorganic nodes, and interfaces on metal-organic frameworks’ (MOFs) thermal conductivity using non-equilibrium molecular dynamics calculations. The study found that the dominance of interface resistance derived from intrinsic framework structures of MOFs, rather than chemical interactions between nodes and linkers. Xing et al. [6] presented a review highlighting the importance of advanced TIMs with high thermal conductivity and low interfacial thermal resistance to enhance heat dissipation performance of high-power electronics. The review introduced different types of TIMs (metal-, carbon-, and polymer-based materials) and their thermal properties, fabrication techniques, developing trends, and it emphasized the need for good thermal and chemical stability, long-term stability, and cost-effectiveness. By summarizing recent advancements in advanced TIMs studies for high-power electronics, the review provided crucial references for designing electronic devices and constructing TIMs for efficient heat dissipation.

Heat transfer via nanofluids has emerged as an effective approach for thermal management in complex systems. Optimization of nano additives and microchannel designs can significantly improve heat transfer efficiency. Apmann et al. [7] investigated the effects of a connector between two microchannels on heat transfer efficiency, using Fe_3_O_4_ nanoparticles as nanofluids. The connector enhanced the heat transfer coefficient within the second microchannel by increasing the randomness of molecules and particles, refreshing the fluid’s memory before entering the second channel. The effects of Reynolds number and nanoparticles on the connector were also studied, revealing that introducing Fe_3_O_4_ nanoparticles increased overall thermal conductivity and the heat transfer coefficient. The connector effectively promoted the random motion of molecules and nanoparticles, with the enhancement being significant at low Reynolds numbers but becoming negligible with increasing Reynolds number. This paper also presented a brief review of current advancements in studying the effects of nanoparticles on fluid thermal conductivity, viscosity, and heat transfer coefficient. Arshad et al. [8] reported on the heat transfer and skin friction rates of various nanofluids over an exponentially stretching surface, using the Rivilin-Erickson tensor and boundary layer approximation theory to construct a mathematical model. They found that the silver-water nanofluid exhibited the optimal heat transfer rate compared to copper-water and zinc-water nanofluids, consistent with previous studies. Their work’s significance lies in the comparative study of the enhanced heat transfer rates and reduced drag and lift coefficients for these three nanofluids over an exponentially stretching surface. The results suggest that the silver-water nanofluid holds promise for use in flat-plate solar collectors and could be tested in natural convective flat-plate solar collector systems under real solar effects.

This Special Issue is expected to be of interest to readerships in both fields of science and engineering. Understanding heat transfer mechanisms in nanostructured materials is fundamental for further developments of nanoscale devices, yet it remains an extreme challenge. We anticipate more emerging works focusing on depicting the underlying physical pictures of heat transfer and developing strategies for thermal manipulation in nanostructures.
